# Takotsubo Syndrome after Pacemaker Implantation: A Systematic Review

**DOI:** 10.31083/j.rcm2312401

**Published:** 2022-12-12

**Authors:** Antonio Strangio, Isabella Leo, Jolanda Sabatino, Letizia Rosa Romano, Claudia Critelli, Giovanni Canino, Ciro Indolfi, Salvatore De Rosa

**Affiliations:** ^1^Department of Medical and Surgical Sciences, Magna Graecia University, 88100 Catanzaro, Italy; ^2^Cardiology Unit, “San Giovanni di Dio” Hospital, 88900 Crotone, Italy; ^3^CMR Unit, Royal Brompton and Harefield Hospital, UB9 6JH London, UK; ^4^Pediatric Cardiology Unit, Department of Children and Woman’s Health, University of Padua, 35128 Padua, Italy

**Keywords:** Takotsubo, pacemaker, procedural complication

## Abstract

**Background:**

Takotsubo syndrome (TTS) is an acute cardiac condition characterized by a temporary wall motion abnormality of the left ventricle that mimics an acute coronary syndrome (ACS). TTS usually occurs following emotional or physical triggering event. More recently, sporadic cases of TTS arising after pacemaker implantation were reported.

**Methods:**

We performed a systematic review of the available literature to provide a comprehensive overview of the current knowledge about pacemaker implantation-induced TTS.

**Results:**

The articles selected included case reports and one registry on 28 patients. Most cases occurred in women (75%), encompassing a broad age range. The mean age of the cases described was 74 years. Full recovery of cardiac function was reported in most cases (92.3%), with largely varying recovery times, on average 7 weeks. The most common comorbidity was arterial hypertension and the average ejection fraction at admission was approximately 62%. Clinical severity ranges from asymptomatic cases to severe clinical heart failure syndrome. Altogether the case fatality rate was 3.6%.

**Conclusions:**

For rare it might be, awareness about the potential to develop TTS after pacemaker implantation should prompt careful clinical monitoring, with daily electrocardiogram (ECG) monitoring and at least one echocardiographic examination prior to patients’ discharge to allow early diagnosis and minimize the clinical risk.

## 1. Introduction

Takotsubo syndrome (TTS) was first described in Japan in the 1990s and is usually characterized by transient wall motion abnormalities of the left ventricle (LV) mimicking an acute coronary syndrome (ACS) [1]. Traditionally, TTS has been typically reported after an emotional or physical trigger event [2]. However, growing clinical experience revealed that several alternative mechanisms can be responsible for TTS, including catecholamine-induced myocardial stunning and inflammation, coronary microvascular dysfunction, and myocardial microinfarction [3, 4], making up a prevalence of 1–3% among all patients presenting with suspected ST-Elevation coronary syndrome [5, 6].

Initially believed of being a very rare condition, it has gradually emerged that several different stimuli can elicit TTS, including pacemaker implantation. The increasing complexity in the assessment of TTS has recently prompted the development of standardized diagnostic criteria, such as the score proposed by the international consensus on TTS (InterTAK Score), as reported in Table 1 [7].

**Table 1.** **Diagnostic criteria proposed by the international consensus on TTS**.

1. Transient left ventricular dysfunction (hypokinesia, akinesia, or dyskinesia) presenting as apical ballooning or midventricular, basal, or focal wall motion abnormalities that usually don’t correspond to a single epicardial vascular distribution. Right ventricular involvement can be also present.
2. The acute event is usually preceded by a stressor (either emotional or physical).
3. Neurologic disorders (particularly vascular disease) as well as pheochromocytoma can also be triggers for takotsubo syndrome.
4. Electrocardiogram (ECG) usually shows new abnormalities (particularly in the ST-T tract).
5. Cardiac biomarkers (troponin and creatine kinase) are usually elevated as well as brain natriuretic peptide.
6. Significant coronary artery disease could also be diagnosed in patients with takotsubo syndrome and does not represent an exclusion criterion for the diagnosis.
7. Patients should have no evidence of infectious myocarditis. Cardiac magnetic resonance could be helpful in distinguish the two conditions.
8. Postmenopausal women represent the majority of takotsubo cases.

In this context, our aim was to provide a comprehensive overview of the current knowledge about pacemaker-induced TTS.

## 2. Methods

For this purpose, we performed a review of the literature on PubMed and Google Scholar. The following keywords have been used for the research: (“takotsubo cardiomyopathy” [MeSH Terms]) OR (takotsubo syndrome [MeSH Terms]) OR (takotsubo pacemaker [Title]) AND (pacemaker [Text Word]) AND (English [Language]). Search results were screened according to the PRISMA protocol by two investigators (AS, IL) independently to identify eligible articles. Divergencies were resolved though discussion on study methodology until consensus was reached. Studies were selected if they fulfilled all the pre-defined inclusion criteria reported. Criteria for inclusion were: (i) original data; (ii) presence of apical ballooning; (iii) exclusion of obstructive CAD; (iv) other cardiomyopathies ruled out or unlikely; and (v) occurrence shortly following pacemaker implantation. Exclusion criteria were: the diagnosis of Takotsubo syndrome wasn’t confirmed nor sufficiently discussed; no relationship with pacemaker implantation was reported; systematic review or meta-analyses. Data extraction was performed by two independent authors (AS, SDR), with divergences resolved by consensus. Baseline characteristics were extracted, including age, gender, cardiovascular risk factors, timing of TTS onset respect to pacemaker implantation, clinical presentation and symptoms, clinical outcome and recovery time.

## 3. Results and Discussion

**Fig. 1.**
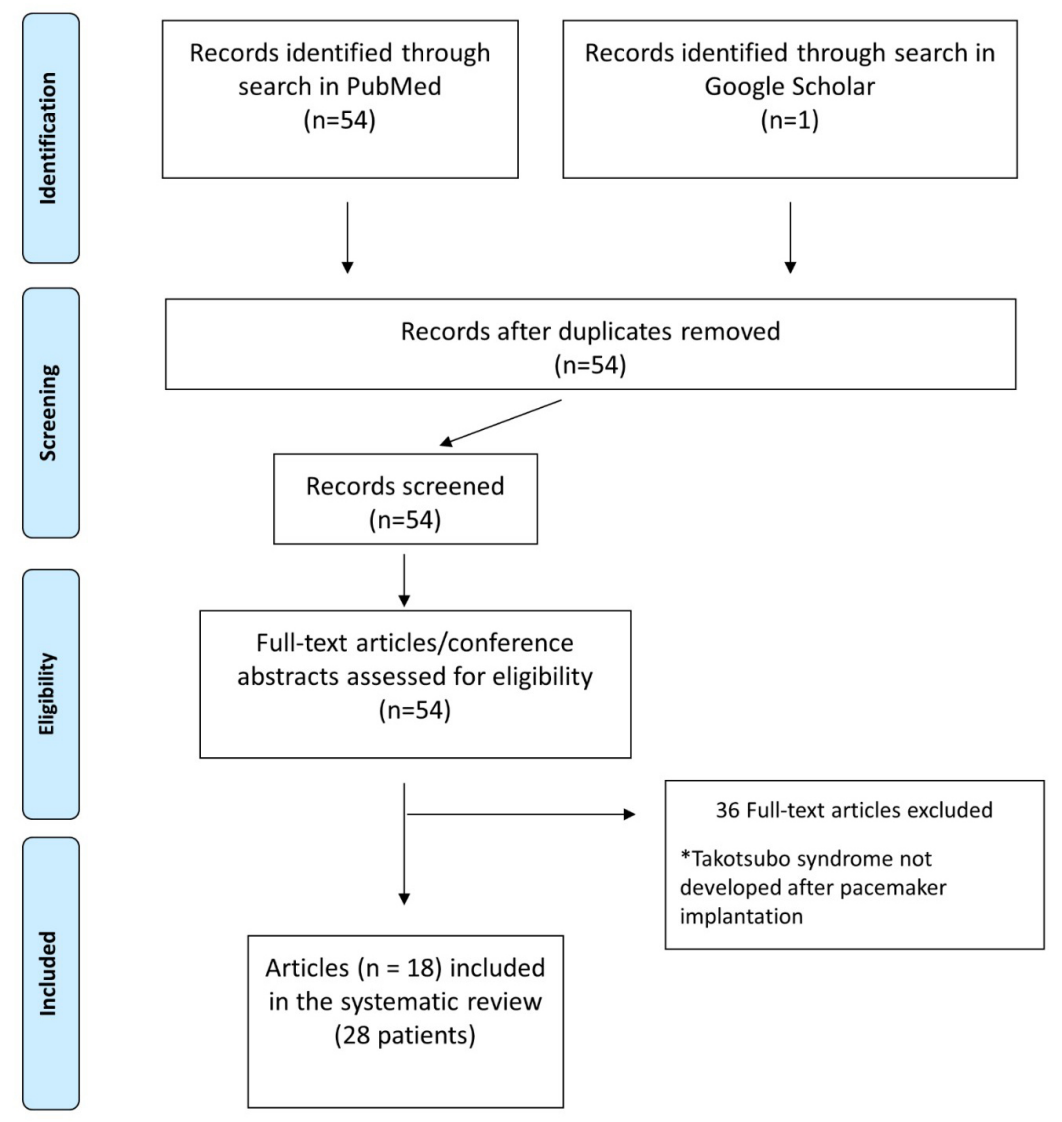
**PRISMA Flowchart describing articles’ screening and selection processes**.

Our search retrieved 54 articles, published between 2006 and 2022. Of those, 18 papers were eligible for inclusion. Thirty-six publications were excluded from the original search. Exclusion criteria are reported in detail in the PRISMA flowchart (Fig. 1) and includecase reports of Takotsubo syndrome not related to device implantation, duplicate studies. The articles selected included 17 case reports and one monocentre registry including altogether 28 patients (21 female and 7 male) that developed TTS after a pacemaker (PM) implantation, mean age 74 years (range 0–91 years), the most common comorbidity was arterial hypertension followed by atrial fibrillation and diabetes mellitus. In almost all the articles included in our review, the only echocardiographic feature reported was the left ventricular ejection fraction (LVEF). In the most case it was in the normal range with few cases that presented a mild reduced LVEF, among the cases where it was reported the mean value was approximately 62% (range 50–75%) [8, 9, 10, 11, 12, 13, 14, 15, 16, 17, 18, 19, 20, 21, 22, 23, 24, 25].

In 19 cases the indication to PM implantation was atrioventricular (AV) conduction defect (AV block) or atrial fibrillation (AF) with slow ventricular response), and Sick Sinus Syndrome (SSS) in the remaining 9 cases. Most of devices implanted were dual chamber PM (n = 23). Clinical details on all cases included are reported in Table 2 (Ref. [8, 9, 10, 11, 12, 13, 14, 15, 16, 17, 18, 19, 20, 21, 22, 23, 24, 25]). Usually, TTS occurs in postmenopausal women and only in 9.7% of cases it affects men [26]. It’s interesting that, in this specific setting, men represent approximately 25% of the population. ECG changes were recorded in almost all cases, except for very few cases [12, 13, 16, 23, 24] in which ventricular pacing probably masked its ECG markers. A recent study [27] has documented that persistent ST elevation (PSTE), defined as ≤50% resolution of peak ST elevation within 48 h from admission, is a relative common characteristic of TTS with a prevalence of 19% and that it is also a prognostic marker. In fact, patients with PSTE had a significantly major number of in-hospital complications and of major adverse cardiovascular events (MACE) at long-term follow-up. Among the cases that we have reported seven presented ST elevation [8, 10, 14, 15, 20] however the authors did not describe if it was persistent. Most patients were symptomatic, with symptoms appearing between the first 10 minutes [8] and up to three days after implantation [18]. However, in about 86% of cases the development of symptoms was recorded in the first 24 hours after PM implantation. Clinical presentation of TTS after PM implantation has been variable, some patients referred dyspnoea, chest discomfort, orthopnoea, nausea, and light-headedness, some debuted with hypotension and, the severe cases presented acute pulmonary oedema and life-threatening arrhythmias. Altogether, six were completely asymptomatic [14, 20, 23, 24], with TTS diagnosed only after routine examinations. For this reason, the real incidence of TTS following pacemaker implantation may be underestimated.

**Table 2.** **Patients’ characteristics and clinical course of all reported cases**.

Authors	Sex	Age	Indication for PM implantation	Type of PM	Clinical presentation	Recovery	Time for recovery	ECG abnormalities	Time to onset of symptoms after PM implantation	Complications of TTS	Death
Kurisu *et al*. [8]	Female	89	AV block	Dual chamber	Chest discomfort	No	Permanent dysfunction	ST-segment elevation in leads I, aVL and V2–6	10 minutes	AHF	No
	Female	77	AV block	Dual chamber	Orthopnea	No	Permanent dysfunction	ST-segment elevation in leads V2–6	3 days	AHF	No
Chun *et al*. [9]	Female	77	SSS	Dual chamber	Asymptomatic	Yes	6 weeks	inverted T waves V2–4	-	No	No
Abu Sham’a *et al*. [11]	Female	86	AV block	Dual chamber	Acute pulmonary edema	Yes	1 week	prolongation of the QTc interval	1 day	Acute pulmonary edema	No
Kohnen *et al*. [10]	Female	83	SSS	Dual chamber	Dyspnea	Yes	9 weeks	ST-segment elevations in the inferior and precordial leads	Few Hours	Left ventricular thrombus	No
Golzio *et al*. [14]	Female	67	AV block	Dual chamber	Chest pain	Yes	12 weeks	ST-segment elevation in V2, T-wave inversion in leads DII-DIII-aVF and V3–V6	1 day	No	No
	Female	64	SSS	Dual chamber	Asymptomatic	Yes	12 weeks	ST-segment elevation in leads DII, DIII, aVF and V2–V6	-	No	No
Brunetti *et al*. [12]	Female	65	AV block	Dual chamber	Dyspnea	Yes	1 week	Paced	Few Hours	AHF	No
Mazurek *et al*. [13]	Male	77	AV block	Dual chamber	Dyspnea	Yes	1 day	Paced	Few Hours	Acute pulmonary edema	No
Gardini *et al*. [15]	Female	75	AV block	Dual chamber	Chest pain and Dyspnea	Yes	Some days	ST segment elevation in inferior and anterior leads.	Few Hours	No	No
Postema *et al*. [16]	Female	61	AV block	Dual chamber	Chest pain and orthopnoea	Yes	3 weeks	Paced	1 day	AHF	No
Kinbara *et al*. [18]	Female	69	AV block	Dual chamber	Chest pain and syncope	-	-	VT	3 days	VT, VF, Acute pulmonary edema	Yes
Dias *et al*. [17]	Female	72	AV block	-	Nausea and lightheadedness	-	-	-	Few Hours	AHF	No
Daswood *et al*. [19]	Female	76	SSS	Dual chamber	Chest pain, Hypotension	Yes	24 weeks	Global deep T wave inversions	1 day	Hypotension, Hypoxia	No
Lazzari *et al*. [20]	Female	67	SSS	Dual chamber	Asymptomatic	Yes	6 weeks	ST-segment elevation of 1 mm in the precordial leads V2-5, rapidly changing to negative T-waves, and widespread repolarization abnormalities	-	No	No
Wei *et al*. [21]	Female	72	AV block	Dual chamber	Chest pain and Dyspnea	Yes	16 weeks	T waves inversion in the pericardial leads	1 day	AHF	No
Wakatsuki *et al*. [22]	Female	81	SSS	Dual chamber	Onset with ventricular tachycardia	Yes	2 weeks	giant negative T waves and a prolonged QT interval	1 day	VT, VF	No
Niewinski *et al*. [23]	Female	75	AV block	Dual chamber	Chest pain	Yes	-	Paced	2 days	No	No
	Male	73	AF with slow ventricular response	Single chamber	Hypotension	Yes	-	LAH	Fews hours	AHF	No
	Female	87	AV block	Dual chamber	Asymptomatic	Yes	-	Paced	-	No	No
	Female	88	SSS	Single chamber	Hypotension	Yes	-	ST segment denivelation	Fews hours	AHF	No
	Male	80	AV block	Dual chamber	Dyspnea	Yes	-	Paced	Fews hours	AHF	No
	Male	75	AF with slow ventricular response	Single chamber	Asymptomatic	Yes	-	ST segment denivelation	-	No	No
	Male	89	SSS	Dual chamber	Asymptomatic	Yes	-	Q-waves	-	No	No
	Female	77	AF with slow ventricular response	Single chamber	Dyspnea	Yes	-	ST segment denivelation and pronlonger QT interval	Fews hours	AHF	No
	Male	92	SSS	Dual chamber	Dyspnea, chest pain	Yes	-	LBBB	Fews hours	Elevation pacing thresholds	No
Iqbal *et al*. [24]	Female	84 days	AV block	Dual chamber	-	Yes	8 weeks	Paced	-	No	No
Moinudddin *et al*. [25]	Male	65	AV block	Dual chamber	Syncope	Yes	4 weeks	Paced/LBB	Fews hours	Hypotension	No

AV block, atrioventricular block; SSS, sick sinus syndrome; AF, atrial fibrillation; VT, ventricular tachycardia; VF, ventricular fibrillation; LAH, left anterior hemiblock; LBBB, left bundle brunch block; AHF, acute heart failure.

Iqbal* et al*. [2] reported the case of a new-born who underwent PM implantation for congenital heart block and hemodynamic instability that developed TTS and apical ballooning early after the procedure. The documented wall motion abnormalities (WMAs) at echocardiography persisted at 20 days follow-up, followed by partial recovery, after starting pharmacological therapy, at 23 days follow-up and complete recovery at ten months of age.

Mean recovery time for LV systolic function among the reported cases was approximately 7 weeks, although Kurisu* et al*. [8] described two cases of persistent LV dysfunction following TTS. Lack of recovery of LV function could be explained either by a too short follow-up period (maximum 4 months) or by an additive potential harmful effect induced by pacing in the context of TTS. Ventricular pacing could in fact cause alterations in electrical and mechanical activation [28, 29, 30, 31, 32]. Data reported in literature showing that chronic pacing at a threshold of 40% pacing burden is cause of LV dysfunction.

In addition, we observed longer recovery times in those cases, compared to the remaining cases described in the literature. Unfortunately, the authors who described the above cases did not report the percentage of ventricular pacing, information that could have made this observation more significant and support the hypothesis of a negative effect on the recovery times of LV function in the setting of TTS in case of a high percentage of ventricular pacing.

LV apical oedema and inflammation, common characteristics in TTS, could be the cause, in the acute setting, of transient dysfunction of the device [33, 34]. Probably myocardial oedema is able to increase local tissue impedance and therefore causing increased pacing threshold, particularly, with bipolar pacing [34, 35]. This hypothesis seems to be supported by the significant correlation between corrected QT interval (QTc) an indirect sign of myocardial oedema, and ventricular pacing thresholds. However, Brunetti *et al*. [36] reported a case of delayed transient ventricular pacing failure in a patient with TTS discharged after partial recovery of LV function, who was referred after 4 weeks with pacing failure, due to a concomitant increase in pacing threshold, subsequently returned within normal ranges after complete LV function recovery. In the case described by Brunetti *et al*. [36] report probably, delayed evidence of pacing failure may coincide with further delayed QT prolongation, which usually peaks in the subacute phase of TTS rather than in the acute phase [37]. Alternatively, it is possible that other conditions beyond QTc prolongation may have influenced ventricular pacing threshold and QT length.

TTS was traditionally considered a benign disease. However, recent data demonstrated that rates of cardiogenic shock and death are comparable to ACS patients treated according to current guidelines [38, 39, 40, 41, 42, 43, 44] and despite long-term follow-up data are missing, the clinical consequences should not be underestimated. In fact, in 4 of the reported cases the patient had haemodynamic instability [9, 14, 23]. Moreover, in other 2 of the reported cases the patient had ventricular tachycardia/fibrillation [22] and of these one died acutely [18].

Interestingly, the absence of symptoms at onset did not always predict a benign course. Indeed, in the case described by Wakatsuki *et al*. [22] the patient was asymptomatic, but she developed polymorphic ventricular tachycardia later. For these reasons, routine investigations are suggested to early identify potentially life-threatening complications even in asymptomatic patients.

Niewinski *et al*. [23] recently conducted a retrospective analysis in one high volume implantation centre, identifying nine cases of TTS following PM, with a prevalence of 0.54% in the analysed period (1655 devices implanted between 2013–2017). In their study, female gender was not predictive of TTS, while the presence of any cognitive decline and frailty syndrome were independent risk factors or TTS occurrence in the described cohort [23].

Evidence-based guidelines for the treatment of TTS are lacking, and current therapeutic strategies are mainly based on clinical experience and expert consensus documents.

**Fig. 2.**
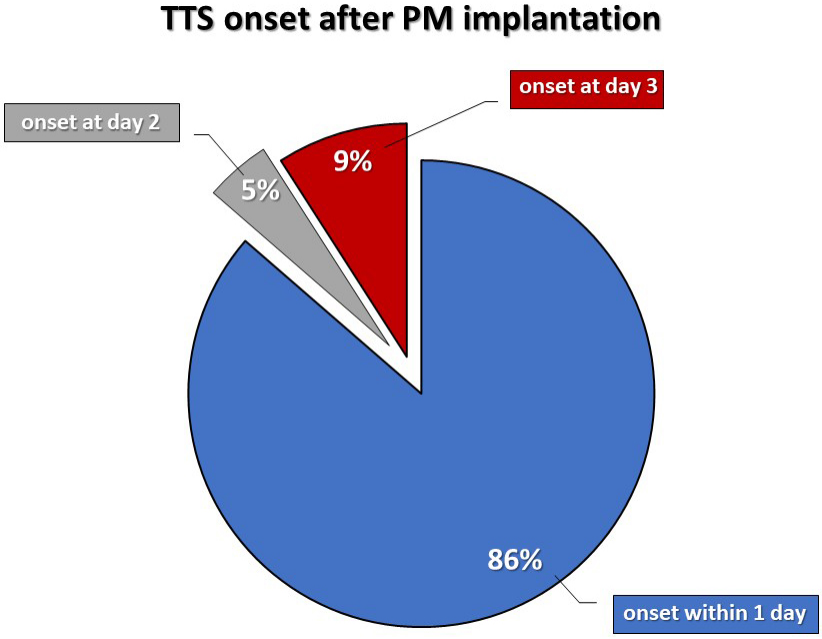
**TTS onset time after pacemaker implantation**.

One open question is whether there might be predicting elements to risk-stratify patients. However, only a multicentre prospective study could provide an answer to this question. Based on currently available information, despite the clinical course was benign in most cases, we should focus our efforts on early diagnosis. In fact, even if symptomatic cases generally presented a benign course, early diagnosis might be crucial in specific cases. What might the most efficient and effective strategy to identify similar cases? Looking at the available data, the onset of TTS was between a few minutes up to three days after device implantation. However, most cases manifested very early after the implantation procedure. In fact, 86% of patients manifested signs or symptoms of TTS within the first 24 hours after PM implantation (Fig. 2). This would render the probability of missing a diagnosis rather low, as a simple fast echocardiogram at discharge would very efficiently identify TTS-related signs. Of course, more solid clinical evidence, ideally from prospective studies is needed to better inform clinical practice in this setting.

## 4. Limitations

The main limitation of this review is that most of the information comes from case reports (17 out of 18 articles) and only one monocentre registry. Therefore, a systematic collection of the clinical and instrumental characteristics of the patients was not performed. Furthermore, no long-term follow-up data are available. However, despite the limited duration of follow up available, the evidence of full recovery of cardiac function in 92.3% of cases is reassuring.

## 5. Conclusions

As pacemaker implantation was recently described as a potential trigger for TTS, we performed a systematic revision of the published literature. Our findings suggest the incidence of TTS after is not so rare as one might think. In fact, the picture showed by our results is a case rate of approximately 0.5%, which could be an underestimation. Furthermore, it is possible that the characteristics of patients affected by TTS after PM implantation reported here do not fully mirror the general population. Several questions still remain open. For example, the typical inflammation and oedema usually found in TTS could be the cause of transient device malfunction. Looking at the clinical course of all clinical cases described so far, daily ECG monitoring and an echocardiogram targeted to wall motion examination prior to patients’ discharge might be helpful to minimize the risk of missing this infrequent, yet dreadful complication of PM implantation.

## Abbreviations

AV block, atrioventricular block; SSS, sick sinus syndrome; AF, atrial fibrillation; VT, ventricular tachycardia; VF, ventricular fibrillation; LAH, left anterior hemiblock; LBBB, left bundle brunch block; AHF, acute heart failure.

## Supplementary Material

Supplementary material associated with this article can be found, in the online version, at https://doi.org/10.31083/j.rcm2312401.
