# Supplementary material for: Takotsubo Syndrome after Pacemaker Implantation: A Systematic Review
Source: Rev Cardiovasc Med. 2022 Dec 12;23(12):401. doi: 10.31083/j.rcm2312401 (PMC11270462; doi:10.31083/j.rcm2312401)
Supplement: Supplementary file 1 [file 2153-8174-23-12-401-s1.pdf]

PRISMA 2020 Checklist

Section and Topic	Item #	Checklist item	Location where item is reported
**TITLE**			
Title	1	Optimal timing of revascularization in patients with STEMI and multivessel disease: a systematic review and meta-analysis.	Title Page
**ABSTRACT**			
Abstract	2	See the PRISMA 2020 for Abstracts checklist.	
**INTRODUCTION**			
Rationale	3	Was to provide a comprehensive overview of the current knowledge about pacemaker-induced TTS	Introduction
Objectives	4	To provide a comprehensive overview or pacemaker-induced TTS.	Introduction
**METHODS**			
Eligibility criteria	5	Criteria for inclusion were (i) original data; (ii) presence of apical ballooning, (iii) exclusion of obstructive CAD (iv) other cardiomyopathies ruled out or unlikely; and (v) occurrence shortly following pacemaker implantation	Methods
Information sources	6	Scientific literature was searched for on the following public databases: PubMed (https://pubmed.ncbi.nlm.nih.gov/) and Google Scholar.	Methods
Search strategy	7	key-words for the search: ((“takotsubo cardiomyopathy” [MeSH Terms]) OR (takotsubo syndrome [MeSH Terms] OR (takotsubo pacemaker [Title])) AND (pacemaker[Text Word]) AND (English[Language])	Methods
Selection process	8	Two reviewers (AS, IL) independently screened search records to identify eligible trials. Divergencies were resolved though discussion until consensus was reached. Manuscripts were selected if they fulfilled all the pre-defined inclusion criteria reported.	Methods
Data collection process	9	Data extraction was performed by two independent reviewers (AS, IL)), with divergences resolved by consensus. Baseline characteristics of the patients included were extracted to an excel worksheet, including age, gender, cardiovascular risk factors, timing of TTS onset respect to pacemaker implantation, clinical presentation and symptoms, clinical outcome and recovery time.	Methods
Data items	10a	The outcomes for which data were sought were: clinical outcome and recovery time.	Methods
	10b	All other variables for which data were sought were: age, gender, cardiovascular risk factors, timing of TTS onset respect to pacemaker implantation, clinical presentation and symptoms	Methods
Study risk of bias assessment	11	NA	
Effect measures	12	NA	
Synthesis methods	13a	NA	
	13b	NA	
	13c	The methods used to tabulate or visually display results of the studies and syntheses have been tables.	
	13d	NA	
	13e	NA	
	13f	NA	
Reporting bias assessment	14	NA	
Certainty assessment	15	The Grading of Recommendations Assessment, Development and Evaluation (GRADE) approach was used to assess the certainty in the body of evidence for all outcomes.	Methods
**RESULTS**			

PRISMA 2020 Checklist

Section and Topic	Item #	Checklist item	Location where item is reported
Study selection	16a	A flow diagram was used to describe the results of the search and selection process, from the number of records identified in the search to the number of studies included in the systematic review.	Figure 1
	16b	Some studies might appear to meet the inclusion criteria, but they were excluded, because they did not report the clinical outcomes related to this topic.	Figure 1
Study characteristics	17	18 manuscripts were case reports, with 28 patients.	Results
Risk of bias in studies	18	NA.	
Results of individual studies	19	NA	
Results of syntheses	20a	NA	
	20b	NA	
	20c	NA	
	20d	NA	
Reporting biases	21	NA	
Certainty of evidence	22	The Grading of Recommendations Assessment, Development and Evaluation (GRADE) approach was used to assess the certainty in the body of evidence for every outcome.	Results
**DISCUSSION**			
Discussion	23a	Our analysis documented similar clinical outcomes with either single-stage immediate complete revascularization and delayed staged complete revascularization, especially when DES are used.	Conclusions
	23b	The study protocol and the PRISMA flowchart are included in the manuscript text.	Main text and supplements
	23c	Only case reports and case-series were found in published literature.	Limitations
	23d	awareness about the potential to develop TTS after pacemaker implantation should prompt careful clinical monitoring, with daily ECG monitoring and at least one echocardiographic examination prior to patients' discharge to allow early diagnosis and minimize the clinical risk	Abstract (page 2) and Conclusions (page 7prospero)
**OTHER INFORMATION**			
Registration and protocol	24a	PROSPERO does not accept scoping reviews, literature reviews or mapping reviews. This should not stop you from submitting your full protocol or completed review for publication in a journal.	Methods
	24b	Protocol details are available in the main text and manuscript supplements	
	24c	NA	
Support	25	No financial or non-financial support for the review was given.	
Competing interests	26	The of review authors have no competing interests.	
Availability of	27	The template of the Prisma Flow Diagram can be found at https://prisma-statement.org/prismastatement/flowdiagram.aspx.	

PRISMA 2020 Checklist

Section and Topic	Item #	Checklist item	Location where item is reported
data, code and other materials			

From: Page MJ, McKenzie JE, Bossuyt PM, Boutron I, Hoffmann TC, Mulrow CD, et al. The PRISMA 2020 statement: an updated guideline for reporting systematic reviews. BMJ 2021;372:n71. doi: 10.1136/bmj.n71

For more information, visit: http://www.prisma-statement.org/
